# Distinct requirements for Tcf3 and Tcf12 during oligodendrocyte development in the mouse telencephalon

**DOI:** 10.1186/s13064-023-00173-z

**Published:** 2023-09-08

**Authors:** Mary Jo Talley, Diana Nardini, Lisa A. Ehrman, Q. Richard Lu, Ronald R. Waclaw

**Affiliations:** 1 Graduate Program in Molecular and Developmental Biology, Cincinnati Children’s Hospital Research Foundation, University of Cincinnati College of Medicine, Cincinnati, OH 45229 USA; 2 Division of Experimental Hematology and Cancer Biology, Cincinnati Children’s Hospital Medical Center, 3333 Burnet Avenue, Cincinnati, OH 45229 USA; 3 Department of Pediatrics, University of Cincinnati College of Medicine, Cincinnati, OH 45229 USA; 4 Division of Developmental Biology, Cincinnati Children’s Hospital Medical Center, 3333 Burnet Avenue, Cincinnati, OH 45229 USA

## Abstract

**Background:**

E-proteins encoded by *Tcf3*, *Tcf4*, and *Tcf12* are class I basic helix-loop-helix (bHLH) transcription factors (TFs) that are thought to be widely expressed during development. However, their function in the developing brain, specifically in the telencephalon remains an active area of research. Our study examines for the first time if combined loss of two E-proteins (Tcf3 and Tcf12) influence distinct cell fates and oligodendrocyte development in the mouse telencephalon.

**Methods:**

We generated *Tcf3/12* double conditional knockouts (dcKOs) using *Olig2*^*Cre/+*^ or *Olig1*^*Cre/+*^ to overcome compensatory mechanisms between E-proteins and to understand the specific requirement for Tcf3 and Tcf12 in the ventral telencephalon and during oligodendrogenesis. We utilized a combination of in situ hybridization, immunohistochemistry, and immunofluorescence to address development of the telencephalon and oligodendrogenesis at embryonic and postnatal stages in *Tcf3/12* dcKOs.

**Results:**

We show that the E-proteins Tcf3 and Tcf12 are expressed in progenitors of the embryonic telencephalon and throughout the oligodendrocyte lineage in the postnatal brain. *Tcf3/12* dcKOs showed transient defects in progenitor cells with an enlarged medial ganglionic eminence (MGE) region which correlated with reduced generation of embryonic oligodendrocyte progenitor cells (OPCs) and increased expression of MGE interneuron genes. Postnatal *Tcf3/12* dcKOs showed a recovery of OPCs but displayed a sustained reduction in mature oligodendrocytes (OLs). Interestingly, Tcf4 remained expressed in the dcKOs suggesting that it cannot compensate for the loss of Tcf3 and Tcf12. Generation of *Tcf3/12* dcKOs with *Olig1*^*Cre/+*^ avoided the MGE morphology defect caused by *Olig2*^*Cre/+*^ but dcKOs still exhibited reduced embryonic OPCs and subsequent reduction in postnatal OLs.

**Conclusion:**

Our data reveal that *Tcf3* and *Tcf12* play a role in controlling OPC versus cortical interneuron cell fate decisions in MGE progenitors in addition to playing roles in the generation of embryonic OPCs and differentiation of postnatal OLs in the oligodendrocyte lineage.

## Introduction

Oligodendrogenesis is a critical event in brain development that results in the generation of oligodendrocyte progenitor cells (OPCs) that differentiate into oligodendrocytes (OLs) which produce myelin that is crucial for the health of the axons [1, 2]. During development, OL generation is under precise spatiotemporal regulation, determined by appropriate activation of signaling pathways and transcription factors (TFs) [3, 4]. Furthermore, single cell RNA sequencing studies have revealed a new level of differential gene expression as neural progenitors, OPCs, and OLs go through distinct stages during brain development [5–7]. Identifying key regulators at these distinct stages is an ongoing effort in the OL field.

The class II basic-helix-loop-helix (bHLH) TFs Ascl1 and Olig2 are regulators early in oligodendrogenesis as knockouts show robust defects in the embryonic generation of OPCs in brain and spinal cord [8–15]. In addition, stage specific conditional knockouts and in vitro cultures revealed that these genes as well as *Olig1* play roles at later stages in postnatal OPC generation and/or OL differentiation [11, 16–21]. These class II bHLH TFs are known to heterodimerize with class I bHLH TFs, called E-proteins, to bind DNA [22–24]. Despite the numerous studies identifying roles for class II bHLH TFs during OL development, the class I bHLH TFs remain understudied in this cell lineage.

Even though E-proteins were thought to be widely expressed, *Tcf4* has been characterized to show enriched regional expression in the developing cortex and hippocampus in the telencephalon [25, 26]. In addition, enriched *Tcf4* and *Tcf12* expression has also been identified in the medial ganglionic eminence (MGE) in the ventral most progenitors in the telencephalon [27, 28]. Previous in vitro studies have revealed that *Tcf3* and *Tcf12* exhibit cell type expression in cultured oligodendrocyte precursors [29]. Additional in vitro studies showed that co-expression of Tcf3 with either Ascl1, Olig1, or Olig2 can influence later stages of OL development [30]. *Tcf12* was also identified as one of the TFs expressed in OPCs derived from human neural progenitor cells [31]. Tcf4 is the most studied E-protein in the developing brain [32]. Loss-of-function mutations of *Tcf4* cause the neurodevelopmental disorder called Pitt Hopkins syndrome and *Tcf4* KO mouse models have revealed roles for *Tcf4* in the cortex and hippocampus [25, 33–36]. In addition, *Tcf4* KOs showed defects in the OL lineage cells, specifically in later OL differentiation stages [37, 38]. Despite these studies on *Tcf4*, there continues to be a lack of understanding of the in vivo requirement for *Tcf3* and *Tcf12* in oligodendrogenesis. In fact, *Tcf3* and *Tcf12* have redundant and compensatory roles and single knockouts revealed no CNS defects [39–43].

We investigated the importance of *Tcf3* and *Tcf12* in OL development by creating a double conditional knockout (dcKO) mouse model using *Olig2*^*cre/+*^ mice. We found a simultaneous loss of *Tcf3* and *Tcf12* resulted in transient abnormal morphology of the MGE, a loss of OPCs, and an increase of MGE interneuron genes during embryogenesis. However, at postnatal stages, OPC numbers recovered from embryonic time points, but mature OL (mOL) populations remained decreased in the dcKOs. Later embryonic deletion of *Tcf3* and *Tcf12* using *Olig1*^*cre/+*^ avoided the MGE phenotype but the embryonic OPC reduction and postnatal OL differentiation defects remained. Our study concludes that *Tcf3* and *Tcf12* play roles in ventral telencephalon cell fate decisions and specifically in the OL lineage for embryonic OPC generation and the generation of postnatal OLs.

## Materials and methods

### Animals

Protocols for animal experiments using mice were approved by the Institutional Animal Care and Use Committee at the Cincinnati Children’s Hospital Medical Center and carried out in accordance with National Institutes of Health guidelines. *Tcf12*^*tm3Zhu*^ ;*Tcf3*^*tm4Zhu*^ (Stock #024511), referred to as *Tcf3/12*^*flox*^ in this manuscript, *Rosa*^*tom/+*^ (Stock #007914), and *FoxG1*^*IRES − Cre/+*^ (Stock #029690) mice were obtained from Jax and genotyped from published protocols on Jax website. *Olig2*^*cre/+*^ mice were previously described [44] and provided by Y. Yoshida. *Olig1*^*cre/+*^ mice were previously described [17]. For specific embryonic collections, vaginal plug indicates embryonic day 0.5 during timed matings. For BrdU treatment, BrdU was dissolved into PBS at a 10 mg/1mL dilution. Pregnant females were administered 0.1 mg of BrdU for every 1 g of weight through intraperitoneal injection, 30 min before embryonic dissection. Embryo and adult collections and processing for histology were completed as previously described [45–47].

### Immunohistochemistry and immunofluorescence

Primary antibodies were used at the following concentrations: Animal-Antibody (Dilution, Company, catalog #), Rbt-Ascl1 (1:5,000 Abcam, #AB74065), Gt-βgal (1:1,000, Biogenesis, #2282), Rbt-βgal (1:1000, Biogenesis, #4600 − 1509), Rat-BrdU (1:250, Abcam, #AB6326), Rbt-CNPase (1:500, Cell Signaling, #5664), MS-CC1 (1:100, Calbiochem #OP80-100UG), GP-Gsx1 (1:5000, [48]), Rbt-Gsx2 (1:4000, [49]), Ch-MBP (1:500, Aves, #AvesMBP), Gt-mCherry (1:1000, Biorbyt, #ORB11618), MS-Neurofilament (1:100, DSHB, #2H3), Rbt-Nkx2.1 (TTF1) (1:2000, Seven Hills Bioreagents, # WRAB-1231), Rbt-Nkx2.2 (1:1000, Abcam, #AB191077), Rbt-Olig2 (1:1000, Millipore Sigma, #AB9610), Gt-Pdgfrα (1:500, R&D Systems, #AF1062), Gt-Sox10 (1:500, Santa Cruz, catalog #SC-17,342), Rbt-Tcf4 (1:500, ProteinTech, #22337-1-AP), Rbt-Tcf12 (1:1000, ProteinTech ,#14419-1-AP). The mouse-neurofilament 2H3 antibody was obtained from the Developmental Studies Hybridoma Bank (DSHB) at the University of Iowa.

Fluorescent stains were cover slipped with SouthernBiotech DAPI Fluoromount-G to stain for DAPI.

In situ hybridization was completed as described [50]. The plasmid to generate *Plp1* anti-sense probe was previously described [11]. The following primers were used to generate in situ probes:

*Lhx6*: Fwd - ATGCACTTCTCACCAGAGGC, Rev w/ T3 sequence - ATTAACCCTCACTAAAGG GGAGACGTCTGACTGCAACA,

*Tcf12*: Fwd - TCTCGAATGGAAGACCGC, Rev w/ T3 sequence - ATTAACCCTCACTAAAGGCTCCCTCCTGCCAGGTTT

*Tcf3*: Fwd – CCCCAACTACGATGCAGG, Rev w/ T3 sequence - ATTAACCCTCACTAAAGGCCGGGCAGAGATATGGTG

*Tcf4*: Fwd - GCGGTCTACGCTCCTTCA, Rev w/ T3 sequence - ATTAACCCTCACTAAAGGTGGGTTCAAGTCAGGGGA

All bright field pictures were captured on a Leica DM2500 microscope with a DMC6200 camera with 1.0x c-mount or DFC500 camera with 0.7x c-mount using Leica Acquisition Software. Fluorescence images were captured on a Nikon C2 confocal microscope using Nikon Elements software. Representative images were selected from at least *n* = 3 animals for each genotype and each stain. “Controls” refer to wild type animals or animals with a mixed status of floxed *Tcf3* and/or *Tcf12* alleles with no Cre, unless otherwise noted.

### Quantification

Immunofluorescent images for quantification in Fig. 2 were taken at 20x magnification with a 1.5x zoom. 4 serial sections of 3 different control animals were examined for each set of E-protein/OL marker stain. Images were taken at 20x for quantification for most stains, with the following exceptions: MBP/NFM (10x), Nkx2.1 (5x for MGE images, BrdU (40x), Tcf4/Olig2 (10x at E15, 20x with 1.5 zoom for P21), Tcf4/DAPI (20x with 1.5 zoom). High magnification images were taken in corpus callosum, unless otherwise specified. For every data set, at least 3 controls and 3 mutants were analyzed. 4 serial sections of the region were imaged for quantification. Raw images were processed through Cell Profiler to identify the number of cells in the image, identify the area of the image occupied by the stains, or stain intensity, as indicated by the graph. Graphs were generated in Prism 9.0 GraphPad.

### Statistics

Differences in controls and conditional double knockouts were assessed using Student’s two-tailed unpaired t-test. Results were considered significant if p-value was less than 0.05. Mean results and p-value are indicated in Results section. Bar graphs represent mean and error bars represent SEM. P-values are also indicated within bar graphs with * = *p* < 0.05, ** = *p* < 0.01, *** = *p* < 0.001 and **** = *p* < 0.0001. Statistical analyses were performed using Prism 9.0 GraphPad.

## Results

### E-protein expression in telencephalon development and oligodendrogenesis

**Fig. 1:**
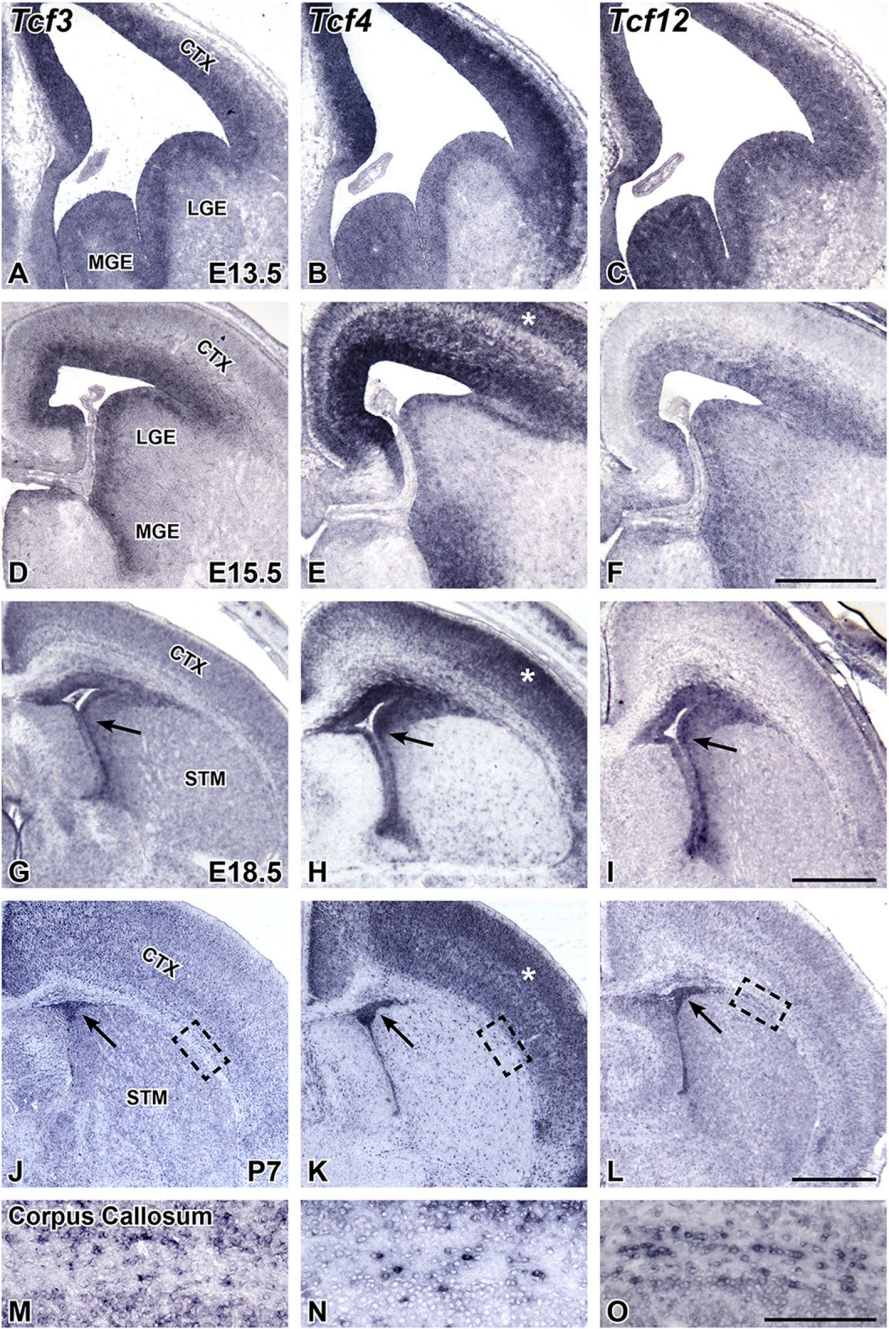
E-protein expression during telencephalon development. In situ hybridization for *Tcf3* (**A, D, G, J, M**), Tcf4 (**B, E, H, K, N**), and *Tcf12* (**C, F, I, L, O**). Representative images are shown at E13.5 (**A-C**), E15.5 (**D-F**), E18.5 (**G-I**), and P7 (**J-O**). At E13.5 and E15.5, all three E-proteins are enriched in the ventricular zone (VZ) in the CTX, LGE, and MGE (**A-F**). In addition, *Tcf4* and *Tcf12* also show high expression within the subventricular zone (SVZ) of the MGE at E13.5 and E15.5 (**B-C, E-F**). Enrichment for the E-proteins in the VZ continues at E18.5 and the SVZ at P7 (see black arrows **G-L**). *Tcf4* specifically is highly enriched in the cortex at E15.5-P7 (see white asterisk, **E, H, K**). High magnification images of the corpus callosum at P7 reveal E-protein expression in the white matter track (**M-O, **black dashed box in** J-L** indicate the representative areas for** M-O**). Scale bar in **F** = 500 μm (for **A-F**), **I** = 600 μm (for **G-I**), **L** = 1 mm (for **J-L**), **O** = 200 μm (for **M-O**). LGE = lateral ganglionic eminence, MGE = medial ganglionic eminence, CTX = cortex, STM = striatum

The genes encoding the three E-proteins were originally thought to be ubiquitously expressed [23, 24]. However, recent research suggests that some of these genes may show distinct regional enrichment during development of the telencephalon. For example, *Tcf4* is robustly expressed in the developing cortex of the dorsal telencephalon, in the hippocampus of the medial telencephalon, and in the globus pallidus of the ventral telencephalon [25–27]. In addition, *Tcf12* expression is enriched in the developing MGE of the ventral telencephalon [28, 51]. However, unlike *Tcf4* and *Tcf12*, *Tcf3* expression is not regionally enriched and is present in both dorsal and ventral progenitors of the telencephalon [52]. We utilized in situ hybridization for *Tcf3*, *Tcf4*, and *Tcf12* at different developmental stages to directly compare E-protein gene expression during development of the telencephalon. The three E-protein genes have clear expression in the ventricular zone (VZ) throughout the telencephalon where the earliest neural progenitors reside at E13.5, E15.5, and E18.5 (Fig. 1A-I). In addition to the VZ expression, *Tcf4* and *Tcf12* are also enriched in the MGE at E13.5 (Fig. 1B, C) which is consistent with previous reports [27, 28]. At E15.5, *Tcf3* and *Tcf12* share similar expression patterns, exhibiting enrichment around the VZ (Fig. 1D,F). At this stage, *Tcf4* begins to take on a distinct expression pattern, specifically enriched in the VZ, MGE, and in the maturing cortical regions (Fig. 1E; see white asterisks for cortex). *Tcf3* and *Tcf12* remain expressed in the VZ (E18.5) and forming subventricular zone (SVZ) at P7 (Fig. 1G, I, J, L, see black arrows for VZ/SVZ). *Tcf4* expression shows the same VZ/SVZ enrichment at E18.5 and P7 as *Tcf3* and *Tcf12*, as well as continued expression in the cortex (Fig. 1H, K, see black arrows for VZ/SVZ) consistent with previous reports [25, 26]. High magnification of the corpus callosum at P7 revealed expression of all three E-protein genes within the forming white matter track where oligodendrocytes are enriched (Fig. 1M-O), which is consistent with previous research [38]. Our comparative expression analysis in the developing telencephalon revealed that all three E-protein genes are widely expressed in the developing VZ/SVZ and in scattered cells of the forming white matter. Interestingly, *Tcf4* and *Tcf12* show regionally enriched expression in the MGE of the ventral telencephalon while *Tcf4* was the only gene that showed robust regional enrichment in the maturing cortex of the dorsal telencephalon.

**Fig. 2:**
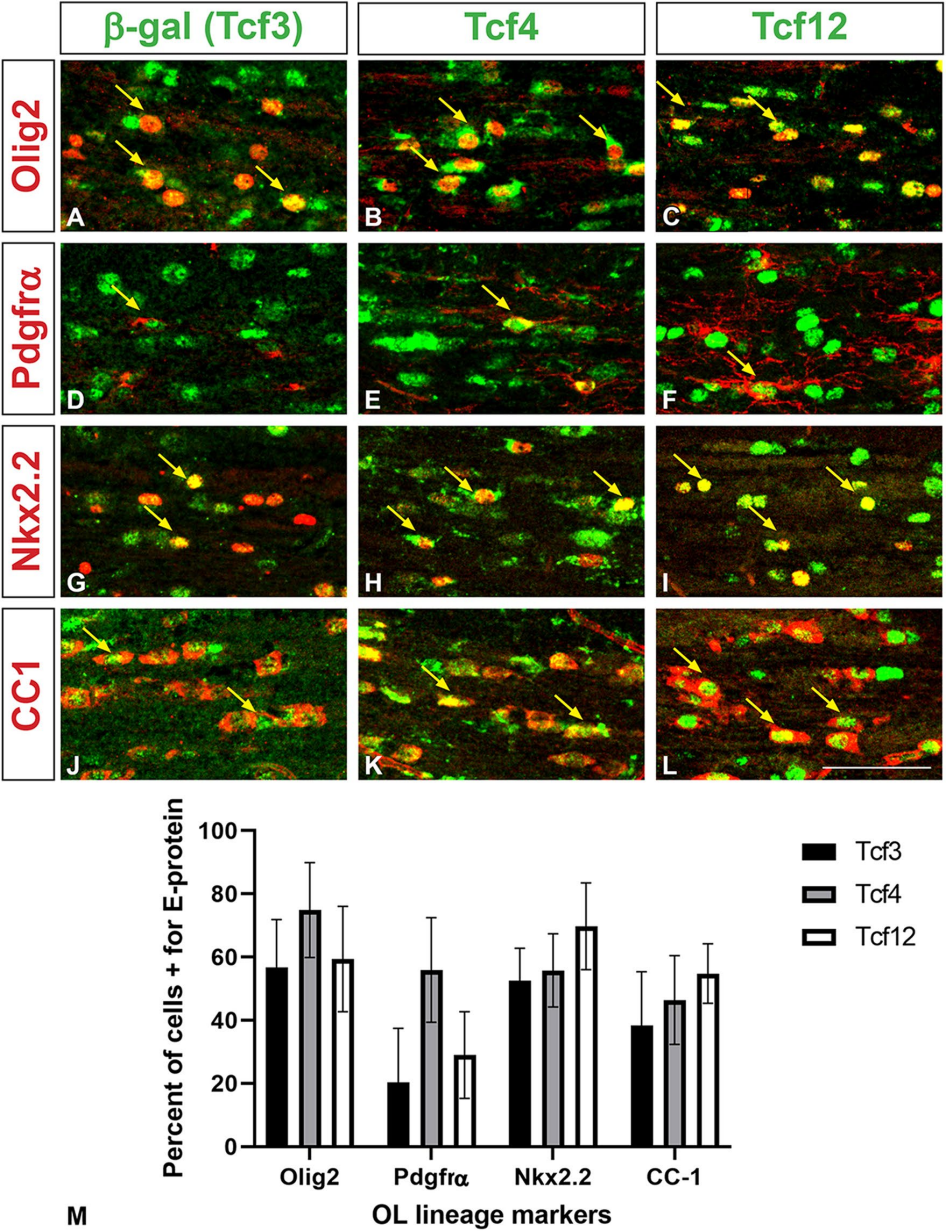
E-protein expression throughout the oligodendrocyte lineage in the corpus callosum. Double immunofluorescence for Tcf4 (**B, E, H, K**), and Tcf12 (**C, F, I, L**) with OL lineage markers. β-gal was used as a reporter for Tcf3 in *Tcf3*^fx/+^;FoxG1^IRES-Cre/+^mice, where lacZ is recombined into frame under the Tcf3 promoter, and double stained for OL lineage markers (**A, D, G, J**). Olig2 was used to mark the entire OL lineage. All three E-proteins have some overlapping expression with Olig2 (see yellow arrows, **A-C**). Pdgfrα was used as a marker for OPC populations (see yellow arrows for overlap with E-proteins, **D-F**). Nkx2.2 marks maturing OPC populations (see yellow arrows for overlap, **G-I**). CC1 was used to define maturing OL populations (see yellow arrows for overlap, **J-L**). Quantification for percentage of OL lineage markers co-expressing the respective E-protein (**M**). Scale bar in L = 50 μm (for **A-L**)

Previous studies have analyzed E-protein gene expression in the oligodendrocyte lineage from RNA sequencing databases and protein expression from in vitro glial cultures [29, 38]. To better grasp the in vivo expression in the postnatal brain and oligodendrocytes, specifically in the scattered cells of the forming white matter, we examined immunofluorescence double stains of the E-proteins and markers of different OL stages. Due to a lack of suitable Tcf3 antibodies for immunofluorescence, a *Tcf3* reporter was used where the *Tcf3*^*fx/+*^ allele has a *lacZ* reporter under control of the *Tcf3* promoter that is out of frame until Cre recombination [53]. We utilized *Tcf3*^*fx/+*^; *FoxG1*^*IRES − Cre/+*^ mice to recombine *lacZ* into frame throughout the telencephalon under control of the *Tcf3* promoter. Control mice were used to examine Tcf4 and Tcf12 expression. Both the *Tcf3*^*fx/+*^; *FoxG1*^*IRES − Cre/+*^ and control mice were analyzed at P21. Double staining with Olig2 and either β-gal (for Tcf3), Tcf4, or Tcf12 in the corpus callosum revealed that all three E-proteins are expressed within the OL lineage, specifically 56.8%±7.51% of Olig2 + cells express Tcf3, 74.9%±7.46% express Tcf4, and 59.4%±8.30% express Tcf12 (*n* = 3) (Fig. 2A-C). Using Pdgfrα as a marker of OPCs, we show that 20.4%±8.50% of Pdgfrα + cells express Tcf3, 55.9%±8.22% express Tcf4, and 29.0%±6.81% express Tcf12 (*n* = 3) (Fig. 2D-F). Nkx2.2 is a marker for OPCs transitioning into pre-myelinating immature oligodendrocytes (iOLs). We show that 52.5%±5.11% of Nkx2.2 + cells express Tcf3, 55.8%±5.76% express Tcf4 and 69.8%±6.80% express Tcf12 (*n* = 3) (Fig. 2G-I). Lastly, CC-1 is used as a marker for maturing iOLs and mOLs. We show that 38.4%±9.06% of CC-1 + cells express Tcf3, 46.4%±7.25% express Tcf4 and 54.8%±4.68% express Tcf12 (*n* = 3) (Fig. 2J-L). These data demonstrate that the E-proteins are expressed at all stages of oligodendrogenesis (Fig. 2M), which is in line with a previous in vitro analysis showing Tcf3 and Tcf12 are expressed in A2B5 + and O4 + rat oligodendrocyte precursors and more recently that Tcf4 is expressed in Pdgfrα+, O4+, and Mbp + cells from cultured rat oligodendroglial cells [29, 38]. In addition, our data is also in line with a searchable sc-RNA seq. gene expression data base at mature adult brain stages showing *Tcf3*, *Tcf4*, and *Tcf12* expression during stages in the oligodendrocyte lineage [5].

### Tcf3/12 dcKOs have deficits in OPC production

**Fig. 3:**
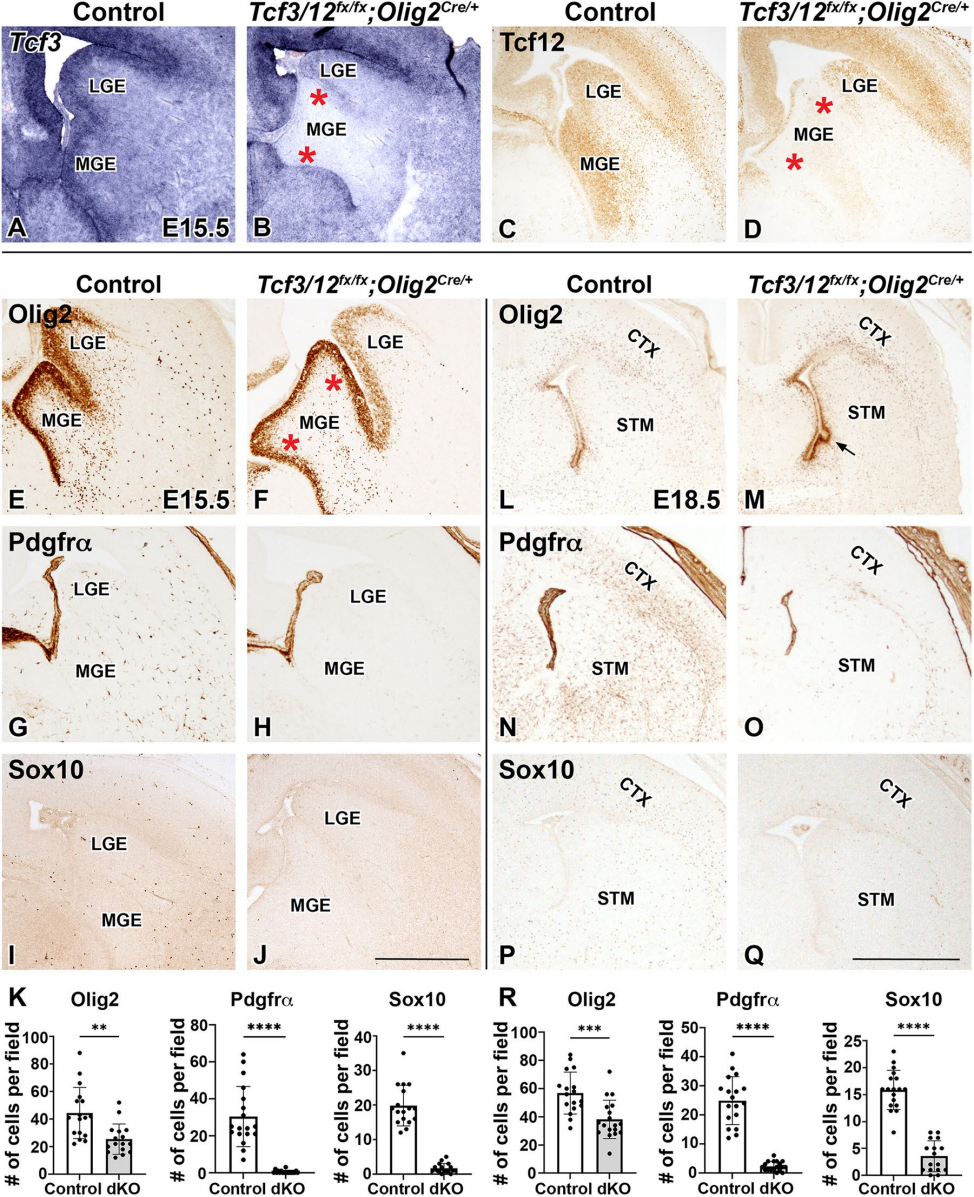
*Tcf3/12* double conditional knock out using *Olig2*^cre/+^results in fewer oligodendrocyte progenitor cells. Confirmation of *Tcf3/12* double conditional KO using *Olig2*^Cre/+^was validated by *in situ* hybridization with probe against *Tcf3* (**A-B**) and immunostaining with an antibody against Tcf12 (**C-D**), showing loss of the respective E-protein gene expression in the double conditional knockouts (dcKOs). Olig2 marks neural progenitor cells in the VZ of the MGE and LGE as well as the OL lineage cells away from the VZ. At E15.5, Olig2 is increased in the VZ of the MGE and decreased in the parenchyma of dcKOs compared to controls (red asterisk shows the increased MGE region, **E-F, K**). Pdgfrα and Sox10 are both markers of OPCs and are largely absent in dcKOs (**G-K**). At E18.5, remnants of the increased MGE Olig2 expression remains visible, while Olig2 expression outside the VZ is decreased in the dcKOs compared to controls (black arrow for MGE remnant, **L-M, R**). Pdgfrα and Sox10 remain severely decreased in dcKOs compared to controls at E18.5 (**N-R**). Quantification of markers at E15.5 in the parenchyma (**K**) and E18.5 in the striatum (**R**). Scale bar in **J** = 500 μm (for **A-J**) and Q = 1 mm (for **L-Q**). LGE = lateral ganglionic eminence, MGE = medial ganglionic eminence, CTX = cortex, STM = striatum

Recent studies have established that *Tcf4* plays a key role in regulating appropriate numbers of oligodendrocyte lineage cells and is required for later stages of OL differentiation and myelination [37, 38]. No requirements for *Tcf3* and *Tcf12* have been identified in the OL lineage. In fact, previous analysis of *Tcf3*^*−/−*^ and *Tcf12*^*−/−*^ knockout mice and *Tcf3/12*^*+/−*^ double heterozygous mice did not find any gross morphological differences in the mutant brains [43]. This is perhaps not surprising because *Tcf3* and *Tcf12* are well described in their ability to compensate for one another in a variety of tissue systems [39–43]. *Tcf3*^*−/−*^, *Tcf12*^*−/−*^, and *Tcf3/12*^*+/−*^ mutations are lethal before breeding age likely from defects in the developing immune system or outside of the CNS, making the generation of a double *Tcf3/12* knockout impossible [43, 54]. Therefore, to examine the cell type specific role for *Tcf3* and *Tcf12* in the developing telencephalon and during oligodendrogenesis without the complication of compensation, we generated a double conditional knockout (dcKO) of *Tcf3/12* using *Olig2*^Cre/+^mice to target deletion in the ventral telencephalon where the majority of embryonic OPCs emerge and through the entire embryonic and postnatal OL lineage. We have previously used this approach to target the RASopathy gene *Ptpn11* in the early oligodendrocyte lineage [47]. Analysis of *Tcf3* gene expression and Tcf12 protein expression confirm robust recombination of *Tcf3* and *Tcf12* alleles in the ventral telencephalon of the dcKOs at E15.5 (Fig. 3A-D).

To assess for the generation of embryonic OPCs from the ventral telencephalon, we stained for the OPC markers Olig2, Pdgfrα, and Sox10 [8, 9, 55–59]. Olig2 is first expressed in the VZ progenitors in the MGE and remains expressed in OPCs of the parenchyma [8, 9, 56]. Pdgfrα and Sox10 are expressed in OPCs and are both downstream of Olig2 [10, 58, 60, 61]. At E15.5, a dramatic reduction of OPC production is observed in the dcKOs compared to controls (Fig. 3E-K). During oligodendrogenesis, Olig2 + progenitors migrate out of the VZ in the ventral telencephalon and begin to express OPC markers Pdgfrα and Sox10 [12, 62]. The dcKOs express Olig2 in the VZ of the MGE, but show a 42.7% loss of Olig2 in the striatum away from the VZ (Fig. 3E-F,K, *p* = 0.0013). At E15.5, dcKOs show a severe depletion of OPCs marked by Pdgfrα and Sox10, with a 98.6% decrease of Pdgfrα (*p* < 0.0001) and 91.4% decrease of Sox10 (*p* < 0.0001) (Fig. 3G-K). This reduction of OPC numbers in the dcKOs continues at later embryonic stages right before birth. The number of Olig2 + cells in the striatum are reduced by 32.7% at E18.5 (Fig. 3L-M,R, *p* = 0.0005). However, Pdgfrα and Sox10 + cells remain severely depleted, with the number of Pdgfrα + cells reduced by 90.8% (Fig. 3N-O,R, *p* < 0.0001) and Sox10 expression reduced by 77.6% (Fig. 3P-R, *p* < 0.0001) in the dcKOs compared to controls. Unlike the robust defect in the *Tcf3/12* dcKO, we did not observe OPC defects analyzing Olig2 + cells in the striatum of *Tcf3* or *Tcf12* single cKOs at E18.5 (Control = 106.7 ± 2.81 compared to *Tcf3* cKO = 95.42 ± 4.01 and *Tcf12* cKO = 94.75 ± 6.01 cells per field, *p* = 0.1350). This is in line with previous studies on germline single knockouts for *Tcf3* or *Tcf12* that did not find obvious CNS defects [43].

Interestingly, Olig2 expression in the VZ of the MGE revealed abnormal morphology in dcKOs at E15.5 (Fig. 3F, see red asterisks for the dorsal and ventral limits of the MGE), which is also apparent in the negative stained regions for *Tcf3* and Tcf12 expression (Fig. 3B, D, see red asterisks for the dorsal and ventral limits of the MGE). This phenotype does persist to some extent at E18.5, where the morphological remnant of the expanded MGE remains apparent (see black arrow, Fig. 3M). Thus, dcKO embryos have a defect in OPC generation or maturation as they maintain expression of Olig2 in ventral progenitor areas and to some extent in the parenchyma cells but the OPC markers Pdgfrα and Sox10, which are expressed after Olig2, are severely reduced at all embryonic stages.

### Postnatal Tcf3/12 dcKOs have reduced markers of mOL

**Fig. 4:**
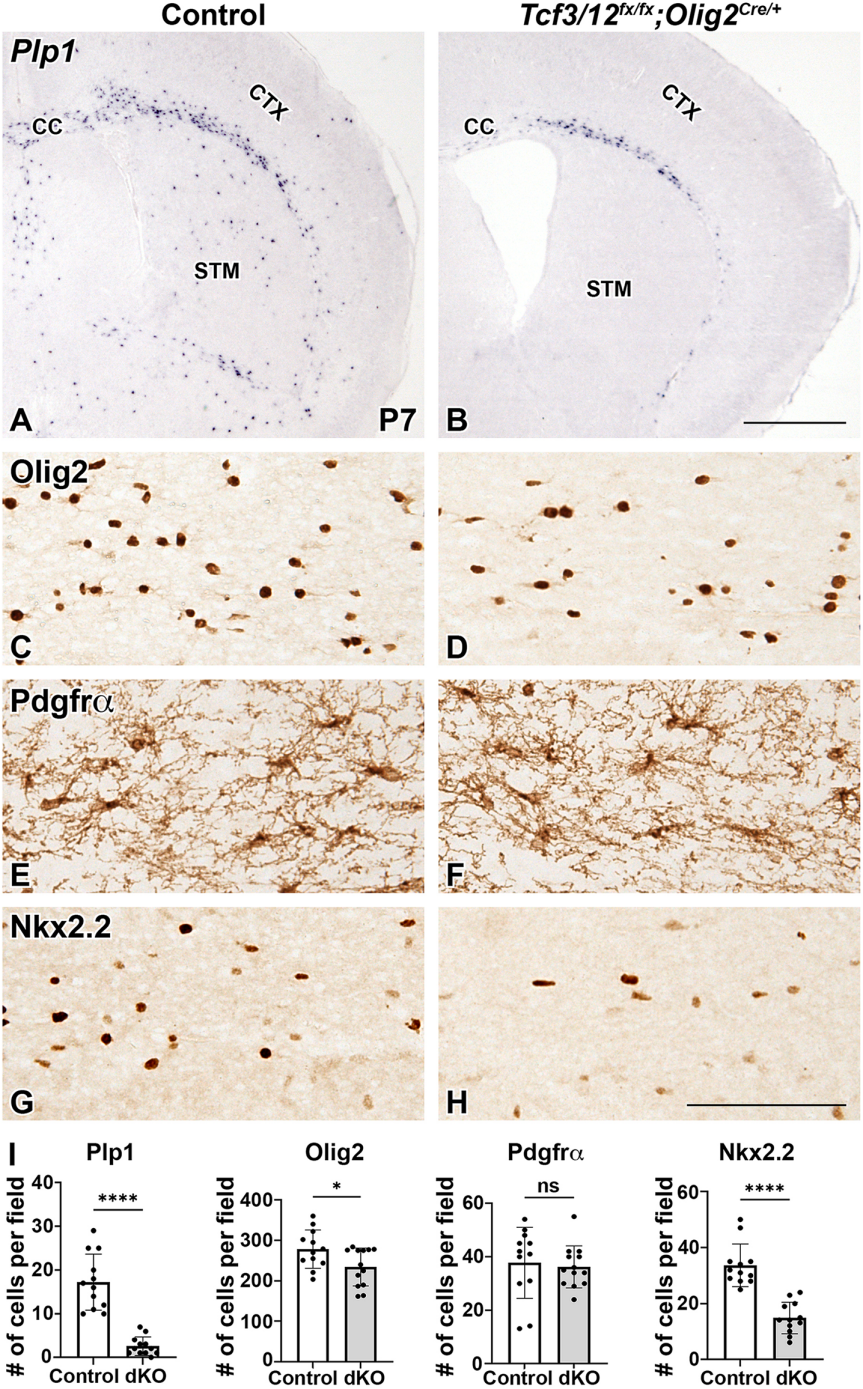
*Tcf3/12* double conditional knock out mice show reduction of mature oligodendrocytes at postnatal time point P7**.** *Tcf3/12* dcKOs have a dramatic reduction of mature oligodendrocytes, marked by *Plp1* gene expression (**A-B, I**). Olig2, marking the entire OL lineage, is also decreased in the CC of dcKOs compared to controls (**C-D, I**). OPC marker, Pdgfrα, shows no difference in expression between controls and dcKOs, indicating a recovery from the E18.5 time point (**E-F, I**). Nkx2.2, expressed in differentiating OPCs, is also decreased at P7 in the dcKOs compared to controls (**G-I**). Images (**C-H**) are high magnification representatives of the corpus callosum in controls and dcKOs. Quantification of respective markers, counted in the corpus callosum (**I**). Scale bar in **B** = 1 mm (for **A-B**) and **H** = 100 μm (for **C-H**). CTX = Cortex, CC= Corpus Callosum, STM = Striatum

Although OPC production is widespread throughout the telencephalon by late embryonic stages, OL differentiation and the expression of maturing myelin markers occurs largely after birth [63]. To determine if *Tcf3/12* dcKOs exhibit OL differentiation defects, we examined the P7 stage as an early time point for gene expression of the myelin marker *Plp1* to label differentiation and Nkx2.2 to label differentiating OPCs [64]. *Plp1* expression can be seen in the corpus callosum and sparsely in the striatum and cortex in controls (Fig. 4A). Therefore, we focused on the corpus callosum/white matter to address the oligodendrocyte markers at this stage. The dcKOs have a severe deficit in *Plp1* positive cells with an 85.0% reduction compared to controls in the corpus callosum and few positive cells were observed in the dcKO striatum or cortex (*p* < 0.0001, Fig. 4A-B, I). Nkx2.2 expression showed a 56.0% decrease in the number of positive cells in the dcKOs compared to controls (*p* < 0.0001, Fig. 4G-H, I). These data suggest that *Tcf3/12* dcKOs exhibit a defect in the production of maturing OLs. Surprisingly, OPCs marked by Pdgfrα recovered to control levels in dcKOs by P7 in the corpus callosum (*p* = 0.7283, Fig. 4E-F, I). The same recovery was also observed in the cortex and striatum which contains more sparsely labeled cells (data not shown). Analysis of Olig2 to label the entire OL lineage (OPCs and OL) revealed only a 15.8% decrease in the dcKOs compared to control, likely reflecting the improvement in OPC numbers and the decrease of the mature cell types (*p* = 0.0277, Fig. 4C-D, I). However, it should be noted that Olig2 also labels a small population of astrocyte progenitors during the first week after birth [65]. Despite this, the Olig2 results are in line with the other oligodendrocyte lineage markers that show in a short time frame from E18.5 to P7, the OPC numbers in the dcKO recover to control levels but significant deficits appear in the differentiation of OPCs to OLs.

**Fig. 5:**
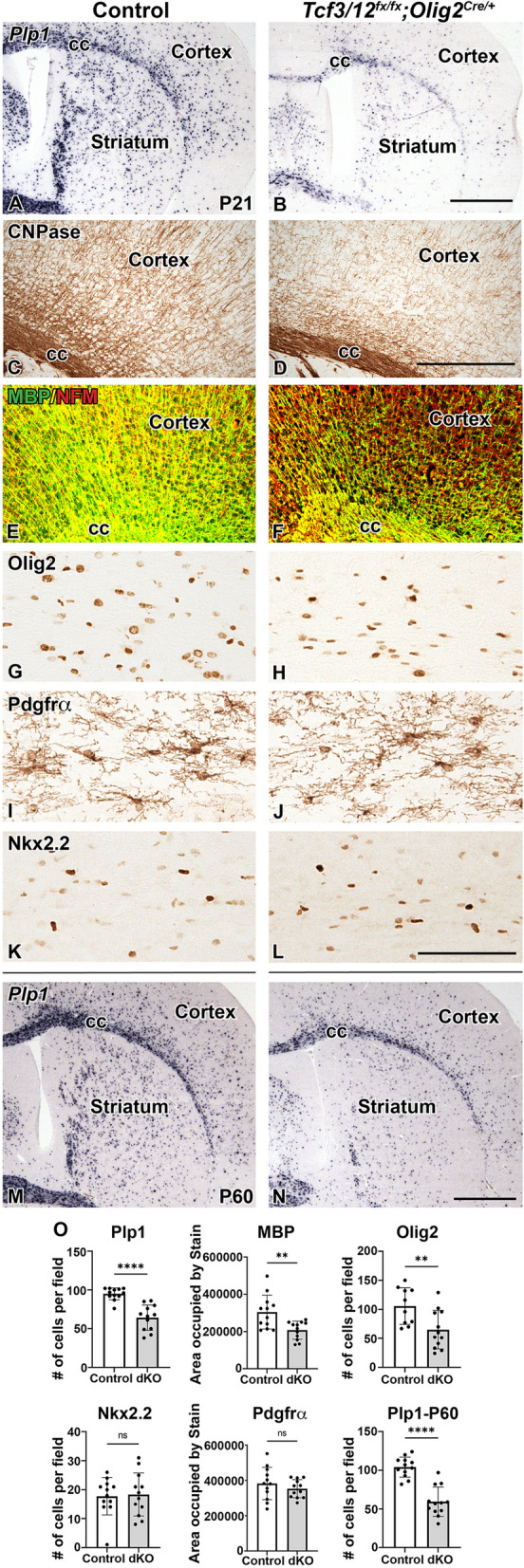
Reduction of mature oligodendrocytes in *Tcf3/12* dcKOs persists at later postnatal stages. At P21, reduction of *Plp1* expression in dcKOs compared to controls is still observed (**A-B,O**). At this stage, protein expression for myelin markers, CNPase and MBP are also reduced in the cortex of dcKOs compared to controls (**C-F,O**).  Note that dcKOs still show expression of neurofilament (NFM) despite the reduction of MBP expression (**E-F**).  As observed in P7, Olig2 is reduced in dcKOs at P21 compared to controls (**G-H,O**) and Pdgfrα+ cell numbers remain similar between the different genotypes (**I-J,O**). At P21, Nkx2.2+ cells are similar in controls and dcKOs (**K-L,O**). To determine if mOL populations continue to improve with age, *Plp1* was examined at P60 and remains reduced in dcKOs compared to controls (**M-O**). Quantification for respective stains counted in the corpus callosum (**O**). Scale bar in **B** and **N** = 1 mm (for **A-B**, **M-N**, respectively), D = 200 μm (**C-F**) and **L** = 100 μm (for **G-L**). CC= Corpus Callosum

The postnatal recovery of OPCs in the dcKO suggests that the OL differentiation defect at P7 may be temporary and the timing of OL generation might be delayed. However, analysis of dcKOs at P21 near the end of developmental myelination revealed the *Plp1* defect was maintained with a 32.5% decrease in dcKOs compared to controls in the corpus callosum (*p* < 0.0001, Fig. 5, A-B,O). At this stage, robust expression of myelin proteins, such as CNPase and MBP, can be observed as an additional method to analyze OL differentiation and the dcKOs show reduce staining density in the cortex (Fig. 5C-F). We detected a 31.8% reduction in MBP expression in the dcKOs compared to control cortical areas, while NFM expression to label the axons is unchanged (*p* = 0.0035, *p* = 0.82 respectively, Fig. 5E-F, O). Despite the deficit in both OL numbers and myelin gene and protein expression, dcKO animals did not exhibit any obvious hypomyelination phenotypes, like shivering or shaking. Similar to the P7 result, there is a decrease in Olig2 + cells labeling the entire OL lineage with a 38.5% reduction in cells at P21 (*p*-value = 0.0085, Fig. 5G-H,O). This decrease likely reflects the reduction in mature OL numbers since OPCs labeled with Pdgfrα were not changed in dcKOs compared to controls (*p* = 0.3430, Fig. 5I-J,O). In contrast to the phenotype at P7, the number of Nkx2.2 + cells is comparable between controls and dcKOs at P21 (*p* = 0.8402, Fig. 5K-L,O). This may indicate a continuing recovery of cells within the OL lineage. However, Nkx2.2 labels mature and differentiating OPCs and newly formed OLs which is a relatively small population at P21, so any reduction in this population might be masked by the existing mature OPCs. To determine if the reduction of *Plp* + cells at P7 and P21 observed in dcKOs is transient and a delay in differentiation, we analyzed 2 month old animals (P60). The reduction in *Plp1* is maintained at these later stages with a 43.0% reduction in dcKOs compared to controls (*p* < 0.0001, Fig. 5M-N,O). This indicates that mOL recovery is not occurring and suggests *Tcf3* and *Tcf12* play a role in the appropriate generation of OLs at postnatal stages, in addition to a requirement for embryonic OPC generation at earlier stages.

### Embryonic Tcf3/12 dcKOs show ventral progenitor abnormalities

**Fig. 6:**
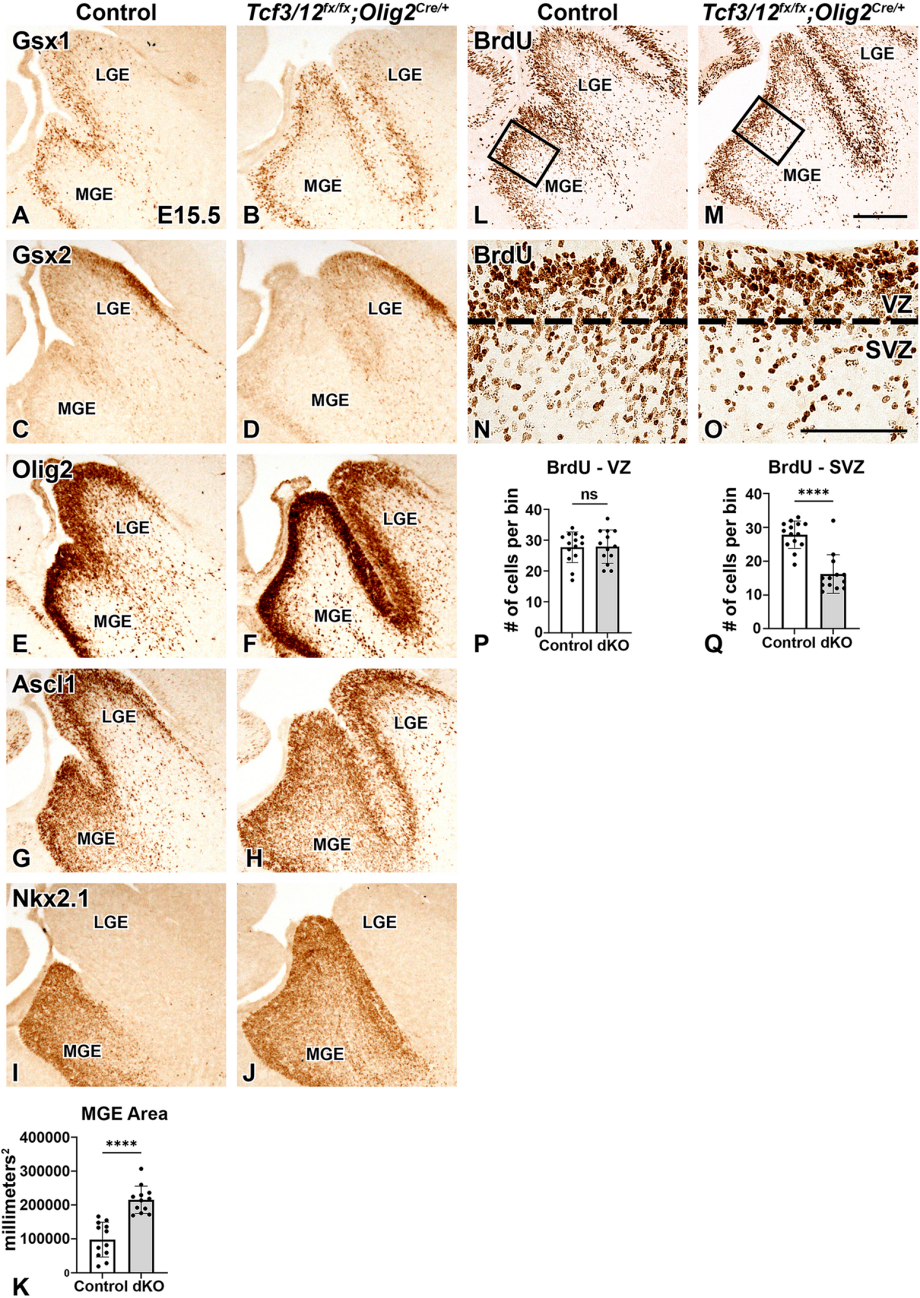
Loss of *Tcf3/12* in the ventral telencephalon results in an increased MGE progenitor region and does not impact regional patterning markers. Multiple markers were used to investigate the different progenitor populations within the MGE: Gsx1 is expressed in progenitors in the VZ in the MGE and LGE (**A-B**), Gsx2 in a high dorsal to low ventral gradient in LGE/MGE progenitors (**C-D**), Olig2 in LGE/MGE progenitors (**E-F**), Ascl1 in LGE/MGE progenitors in the VZ and SVZ (**G-H**), and Nkx2.1 is expressed in progenitors defining MGE boundaries (**I-J**). Expression of all MGE progenitor populations examined show expression spanning the larger MGE in the dcKOs, while general patterning is maintained. MGE area quantified by area of Nkx2.1 expression (**K**). BrdU pulses were performed 30 minutes before E15.5 dissection (**L-M**). Boxes in **L** and **M** refer to approximate areas for high magnification shown in **N** and **O**.  There was no difference in the number of BrdU+ cells in the VZ when controlling for MGE size, but a decrease in BrdU+ cells in the SVZ of the MGE (**N-O**, quantification **P,Q**). Scale bars M = 200 μm (for **A-M**), **O**= 100 μm (for **N-O**). MGE = medial ganglionic eminence, LGE = lateral ganglionic eminence, STM = Striatum

Upon analyzing Olig2 expression and the OPC phenotype in dcKOs at E15.5, it became apparent that dcKOs also exhibit a ventral morphological phenotype observed by Olig2 expression in MGE progenitors (see asterisk Fig. 3E-F and higher magnification images in Fig. 6E-F). The area of the MGE is dramatically increased in the dcKOs compared to controls (120.2% increase in area of Nkx2.1 expression, *p* < 0.0001, Fig. 6A-K). To better understand this phenotype in the ventral progenitors in *Tcf3/12* dcKOs and characterize the increased MGE region, we analyzed the E15.5 stage for patterning and proliferation markers since dcKO MGE phenotype largely resolves by E18.5 (Fig. 3) and that OPCs are actively generated from the MGE and LGE around the E15.5 stage [66]. Therefore, we could get an assessment of general patterning and progenitors in the dcKO. To accomplish this, we analyzed Gsx1, Olig2, and Ascl1 progenitor genes that are expressed in VZ/SVZ cells of the MGE and LGE. In addition, we examined Gsx2, which shows high expression in the LGE and low expression in the MGE and Nkx2.1, which shows specific expression in the entire MGE to address regional patterning in the dcKOs. The expression of these developmental markers reveals that the MGE is expanded in the dcKOs but the overall patterning in the ventral telencephalon is similar to controls suggesting no obvious patterning defect despite the enlarged MGE (Fig. 6A-J). We next examined cell proliferation in MGE progenitor cells at E15.5 to address a stage where the *Tcf3/12* dcKO embryos have severely reduced OPC markers and expanded MGE region. Pregnant mice were injected with BrdU 30 min before E15.5 embryonic dissection to identify cells in S-phase. BrdU expression in the VZ of the MGE show similar numbers of proliferating cells per area in controls and dcKOs, with the area of the VZ being larger in the dcKOs (Fig. 6L-Q). This suggests that while dcKOs have more BrdU + cells in the VZ due to the enlarged MGE, the similar numbers of BrdU + cells per area (controlling for size) may indicate the VZ cells are not proliferating at a greater rate at the E15.5 stage. When analyzing the SVZ directly below the VZ at this stage, however, the number of proliferating cells/mm^2^ is significantly decreased with the dcKOs showing a 42.0% decrease compared to controls (Fig. 6L-Q, *p* < 0.0001). Therefore, even though the MGE region is larger at E15.5 in dcKOs, defects exist in the number of progenitors proliferating in the secondary progenitor area (SVZ). In addition, this data shows the enlarged MGE SVZ/Mantle region in dcKOs is not occupied by an increase in proliferating cells.

**Fig. 7:**
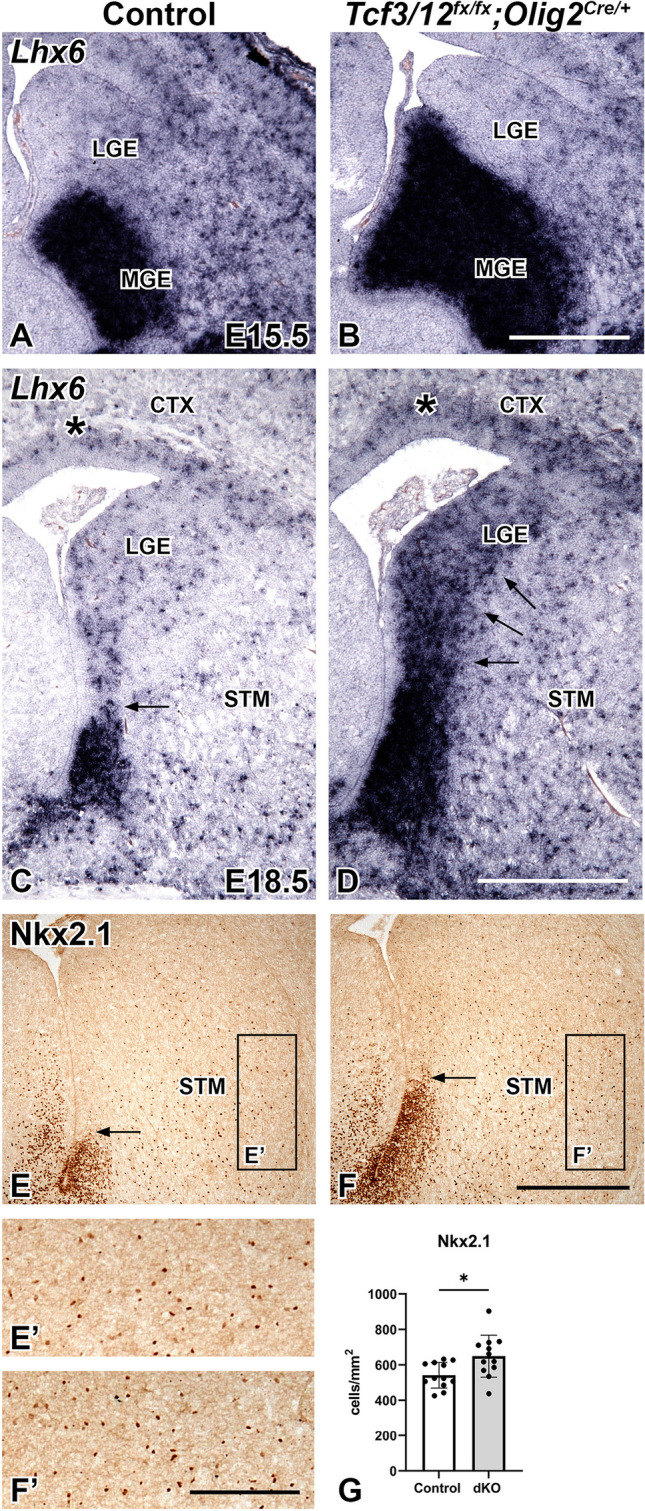
Loss of *Tcf3/12* in the ventral telencephalon results in increased neuronal markers. At E15.5 and E18.5, *Lhx6* expression was examined in the ventral telencephalon to study cells committed to the interneuron fates (**A-D**). The increased MGE in the dcKOs at E15.5 is occupied with *Lhx6* expression (**B**). At E18.5, the dcKOs continue to show increased *Lhx6* expression in the MGE remnant and along path that the interneurons use to migrate to the cortex in the SVZ of the LGE (**C-D**). Striatal interneurons, determined by Nkx2.1 expression, shows increased numbers in the dcKOs compared to controls at E18.5 (**E-F', **Quantification** G**).  Boxes in **E** and **F** represent approximate high magnification areas in **E’** and **F’**. Scale bars **B** = 500 μm (for **A-B**), **D** = 500 μm (for **C-D**), **F** = 500 μm (for **E-F**), **F’** = 200 μm (for **E’-F’**). MGE = medial ganglionic eminence, LGE = lateral ganglionic eminence, CTX = Cortex, STM = Striatum

To determine if the expanded Nkx2.1 MGE domain in *Tcf3/12* dcKOs results in neuronal defects, we analyzed embryonic markers of cortical and striatal interneurons from the MGE. Lhx6 is a downstream target of Nkx2.1 in the MGE and is expressed in developing interneurons [28, 67–70]. *Lhx6* is expressed in the enlarged MGE region of the dcKOs at E15.5 (Fig. 7A, B). Interestingly, dcKOs also show increased *Lhx6* staining in the SVZ of the LGE and cortex at E18.5 (Fig. 7C, D), along the path the interneurons use to migrate to the cortex [69]. Moreover, E18.5 dcKOs also showed expanded ventral progenitor domain of Nkx2.1 (compare arrows in Fig. 7E, F) and a 19.9% increase of Nkx2.1 + striatal interneurons compared to controls (*p* = 0.013, Fig. 7E’,F’,G). Interestingly, the expanded staining for Lhx6 and Nkx2.1 along the migratory path in the LGE region at E18.5 was not apparent earlier at E15.5 (Figs. 6 and 7). Our data show that loss of *Tcf3/12* increases the area of the MGE progenitor domain which subsequently leads to increased *Lhx6* and Nkx2.1 + cells near the SVZ of the LGE and also increased Nkx2.1 + striatal interneurons. Combined our data suggests that *Tcf3* and *Tcf12* play a key role in regulating the interneuron oligodendrocyte fate choice in the embryonic ventral telencephalon.

### Tcf4 expression is preserved in the dcKOs

**Fig. 8:**
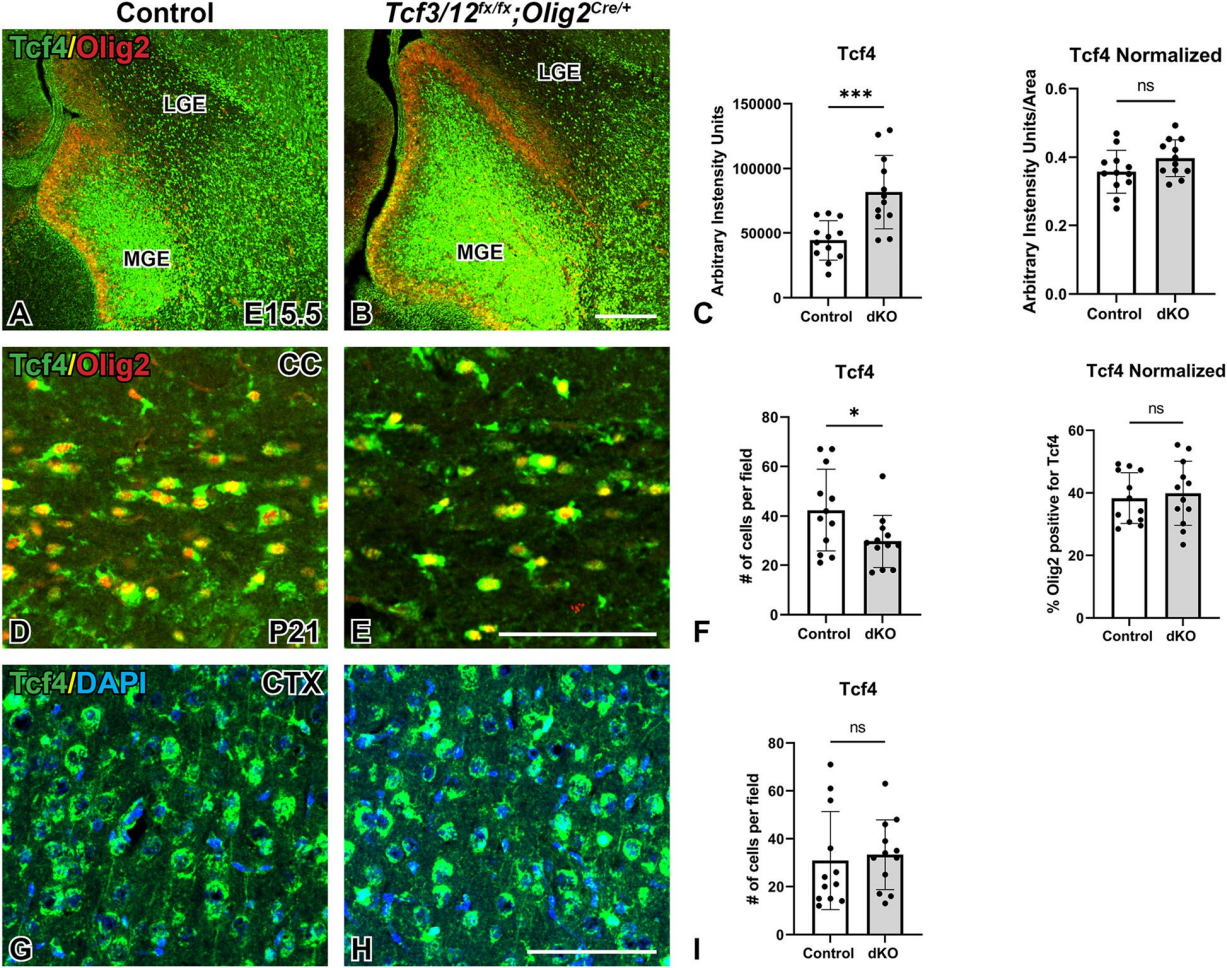
Tcf4 is expressed in *Tcf3/12* dcKOs. At E15.5, Tcf4 is expressed within the MGE in controls (**A**). This expression is also seen in the dcKOs, although more Tcf4 expression is seen due to increased MGE size (**B**). However, when controlling for size of the MGE, there is no difference in Tcf4 expression (Quantification for total intensity and intensity normalized to area - **C**). At P21, less Tcf4+ cells are detected in the corpus callosum in dcKOs compared to controls (**D-E**). Since there are overall fewer OLs in the corpus callosum, Tcf4 expression was normalized to percentage of Olig2+ cells co-expressing Tcf4. Once normalized, Tcf4 expression had no significant difference between controls and dcKOs (Quantification for average cell numbers and normalized percentage – **F**). Tcf4 is highly expressed in the cortex under normal conditions, and no difference is observed between controls and dcKOs (**G-H**, Quantification – **I**).  Scale bars **B** = 200 μm (for **A-B**), **E** = 100 μm (for **D-E**), and **H**= 100 μm (for **G-H**)

Our conditional knockout approach deletes *Tcf3* and *Tcf12* in both VZ progenitors in the ventral telencephalon and oligodendrocyte lineage. However, the remaining E-protein gene, *Tcf4*, could compensate for the loss of *Tcf3/12*. In fact, a recent study has revealed that *Tcf4* is expressed in maturing OLs and plays a key role for the appropriate timing of OL differentiation [38]. Therefore, we analyzed Tcf4 protein expression in embryonic and postnatal dcKOs. Tcf4 is enriched in the MGE and ventral telencephalon at E15.5 (Figs. 1E and 8A). In line with the expanded MGE region, *Tcf3/12* dcKOs show an 84.2% increase of Tcf4 expression in the MGE (*p* = 0.0006, Fig. 8A-C). However, there is no difference in Tcf4 expression in the controls and dcKOs when controlling for the increased MGE size in dcKOs, (*p* = 0.11, Fig. 8A-C). Therefore, the pattern of Tcf4 expression is similar in controls and dcKOs. We next analyzed the oligodendrocyte-rich corpus callosum region at postnatal stages. There is a 29.9% decrease of Tcf4 + cells in the corpus callosum at P21 in the dcKOs (*p* = 0.0365, Fig. 8D-F). However, there is a decrease of OLs in the corpus callosum (Fig. 5) and therefore less total OLs cells in the area. Thus, the percentage of Olig2 + cells in the corpus callosum in the dcKOs that are positive for Tcf4 is the same between controls and dcKOs (*p* = 0.69, Fig. 8D-F). There was no difference in Tcf4 expression detected between controls and dcKOs in the postnatal cortex (*p* = 0.74, Fig. 8G-I) either. Our data indicates that Tcf4 remains expressed in the MGE and oligodendrocyte lineage of *Tcf3/12* dcKO and is unable to fully compensate for the loss of *Tcf3* and *Tcf12* even if it has any redundant role.

### Conditional deletion of Tcf3/12 using Olig1^Cre/+^ leads to OL lineage defects

**Fig. 9:**
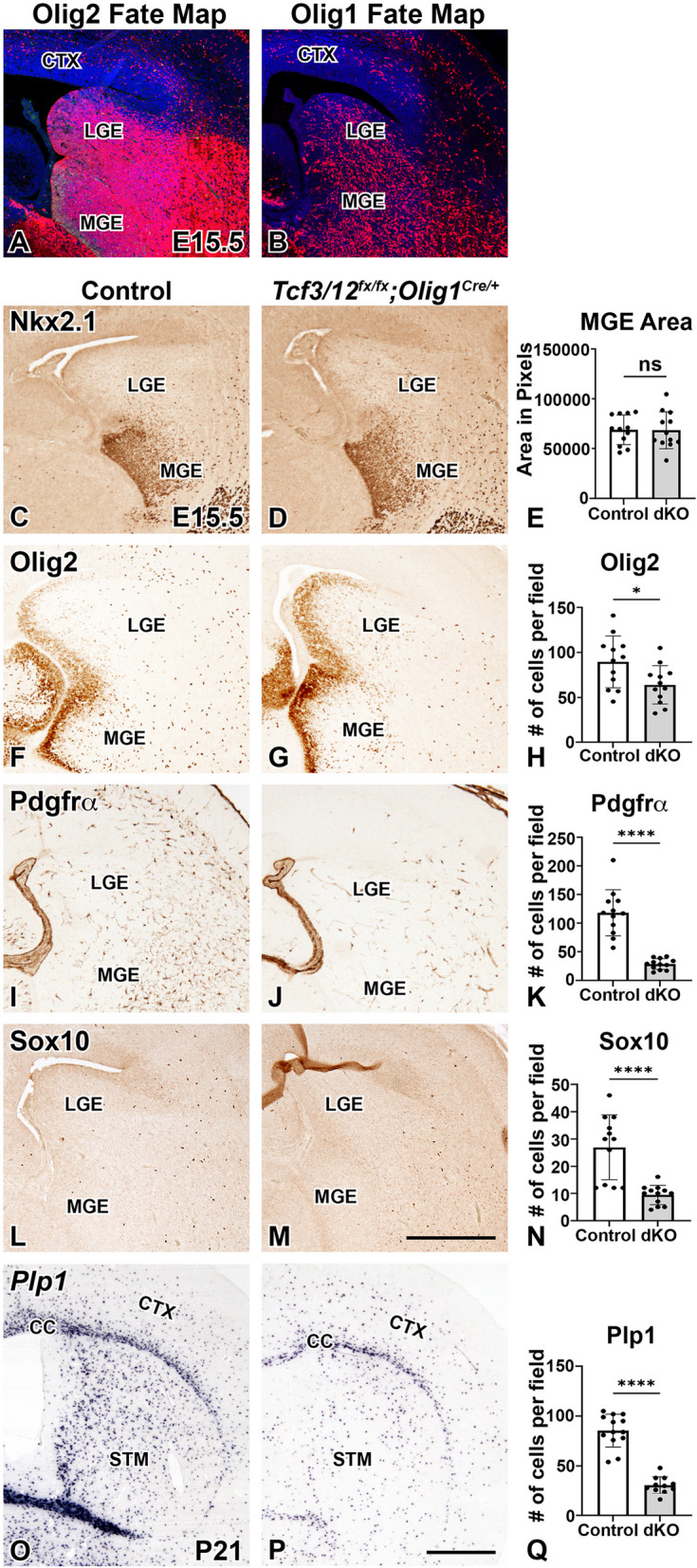
Conditional knock out of* Tcf3/12* using *Olig1*^cre/+^results in similar oligodendrocyte defects, without the MGE phenotype observed in *Tcf3/12* dcKOs generated with  *Olig2*^Cre/+^. Fate map of *Olig2*^Cre/+^using *Rosa*^tom/+^as a reporter of Cre activation shows recombination throughout the MGE and LGE and cells migrating to the cortex (**A**). Fate map of *Olig1*^Cre/+^using *Rosa*^tom/+^ shows later recombination in the ventral progenitors and is enriched in OPCs (**B**). *Olig1*^Cre/+^ driven conditional knockout *Tcf3/12* preserves MGE size in the dcKOs, as marked by Nkx2.1 expression (**C-E**). Similar to *Tcf3/12* dcKOs generated with *Olig2*^Cre/+^, *Tcf3/12* dcKOs generated with *Olig1*^Cre/+^have a reduction of Olig2 expression in the parenchyma compared to controls at E15.5 (**F-H**). *Tcf3/12* dcKOs generated with *Olig1*^Cre/+^also have a dramatic reduction in Pdgfrα+ and Sox10+ OPCs compared to controls at E15.5 (**I-K** and **L-N**, respectively) and reduced numbers of *Plp*+ cells compared to controls at P21, (**O-Q).**Scale bars **M**= 500 μm (for **A-M**) and **P**= 1 mm (for **O-P**)

We are unable to distinguish if the embryonic OPC or postnatal OL phenotypes in the *Tcf3/12* dcKOs is related to the ventral progenitor or interneuron phenotype observed in the dcKOs or if *Tcf3* and *Tcf12* have a specific role within the OL lineage. To address this, we generated dcKOs with *Olig1*^*Cre/+*^, which recombines later then to *Olig2*^*Cre/+*^ (Fig. 9A-B) in the ventral telencephalon. Unlike *Olig2*^*Cre/+*^ that drives robust recombination in nearly all MGE progenitors and some LGE progenitors [48], *Olig1*^*Cre/+*^ does not robustly target these cells and is highly enriched in OPCs and the subsequent OL lineage cells [17]. *Tcf3/12* dcKOs generated with *Olig1*^*Cre/+*^ do not exhibit the abnormal ventral MGE phenotype as Nkx2.1 staining is similar in controls and dcKOs at E15.5 (*p* = 0.9557, Fig. 9C-E). Nevertheless, there remains a decrease in the OPC markers Olig2 (28.6%, *p* = 0.0217), Pdgfrα (75.7%, *p* < 0.0001), and Sox10 (65.0%, *p* < 0.0001) in the *Olig1*^*Cre/+*^ driven dcKOs (Fig. 9F-N). In addition, *Tcf3/12* dcKOs generated with *Olig1*^*Cre/+*^ display reductions in mature OLs as labeled by *Plp1* expression similar to the dcKOs generated with *Olig2*^*Cre/+*^. The *Olig1*^*Cre/+*^ dcKOs have 64.0% less *Plp1* + cells compared to controls at P21 (*p* < 0.0001, Fig. 9O-Q). This indicates that *Tcf3* and *Tcf12* play a role in embryonic OPC generation and postnatal OL differentiation that is independent of the MGE phenotypes observed in *Tcf3/12* dcKOs generated with *Olig2*^*Cre/+*^. Collectively, our data suggests that these two E-protein genes play multiple roles during development in the generation of appropriate numbers of OPCs and cortical interneurons in the embryonic telencephalon and the differentiation of OLs after birth.

## Discussion

This study examines the role of *Tcf3* and *Tcf12* during OL development. Our conditional loss-of-function studies reveals that *Tcf3* and *Tcf12* play roles in the development of ventral progenitors in the MGE by regulating the interneuron and OPC fate choice, and also in the OL lineage for the generation of embryonic OPCs and the appropriate numbers of maturing postnatal OLs. These phenotypes are observed even though the remaining E-protein, Tcf4, continues to be expressed in the embryonic (MGE) and postnatal (OLs) populations effected by *Tcf3/12* loss. Moreover, we found the reduction of embryonic OPCs and postnatal OLs in dcKOs occurs in the absence of the MGE phenotype by using a conditional knockout approach to enrich for deletion in the OPC/OL lineage using *Olig1*^*cre/+*^. Overall, data from our study uncover the importance for *Tcf3* and *Tcf12* in progenitors of the ventral telencephalon and during oligodendrogenesis.

The *Tcf3/12* dcKOs show a dramatic phenotype in the loss of OPCs labeled by Sox10 and Pdgfrα in the telencephalon during embryonic development. In line with this, loss of class II bHLH TFs Ascl1 and Olig2, potential binding partners for E-protein, results in severe reduction of embryonic OPCs [11, 12, 58, 71]. One possible explanation for the loss of OPCs in the dcKOs could be a result of disrupting the class II function in OPC production by removal of the class I bHLH binding partners (Tcf3 and Tcf12). Recent biochemical studies revealed that Tcf4 has interaction with Olig1, Olig2, and Ascl1, Tcf12 interacted with Olig1 and Ascl1, and Tcf3 interacted with Olig2 and Ascl1 [38]. It is striking that the OPC phenotype in our model is unique from studies of *Tcf4* KOs that show no perturbations of OPCs in the spinal cord or the ex vivo telencephalon cultures but robust defects in OL differentiation [38]. Therefore, it seems possible that combined *Tcf3* and *Tcf12* function in OPC generation during early stages where as *Tcf4* functions to regulate the timing of OL differentiation at later stages. Another recent study showed enhanced survival of OPCs that migrate to the olfactory bulb in *Tcf4* conditional mutants generated with *Nkx2.1-cre* suggesting there also may be region specific effects in the CNS for E-protein loss in the CNS [72]. However, our study revealed that Tcf4 remains expressed in the ventral regions of *Tcf3/12* dcKOs suggesting its inability to fully compensate for the loss of *Tcf3* and *Tcf12* during OPC generation even if Tcf4 is involved in OPC survival.

*Tcf3*^*−/−*^ and *Tcf12*^*−/−*^ mice showed similar lethal phenotypes to *Tcf3/12*^*+/−*^ mice, suggesting E-protein dosage is more important than E-protein identity [43]. Importance of bHLH gene dosage has also been demonstrated in OPCs, as *Ascl1/Olig2*^*+/−*^ show more severe phenotypes than single *Ascl1*^*+/−*^ and *Olig2*^*+/−*^ mice [12]. Also, *Tcf4/Olig2*^*+/−*^ showed similar OL differentiation phenotypes to *Tcf4*^*−/−*^ which suggests a key interaction of Tcf4 and Olig2 in OLs [38]. In our experimental approach we conditionally deleted all four alleles of *Tcf3/12*. In addition, both *Olig2*^*Cre/+*^ and *Olig1* ^*Cre/+*^ are knock-in knock-out Cre driver mice, so only one functional allele for the gene is present, further reducing the bHLH TF dosage in our *Tcf3/12* dcKOs. Given the recent finding that Tcf12-Olig1 and Tcf3-Olig2 interaction partners exist [38], future studies are required to understand whether the phenotypes in the *Tcf3/12* dcKOs are due to compound dosage effects specifically with *Olig1* or *Olig2* or if *Tcf3/12* are distinctly required in embryonic production of OPCs.

The enlarged MGE in the *Tcf3/12* dcKO is a phenotype that has not been reported in any other bHLH knockout model. In fact, loss of *Ascl1* results in the opposite phenotype with reduced MGE progenitors and a near complete absence of a pronounced ventral most eminence region [73]. This suggests that the MGE phenotype in *Tcf3/12* dcKO is not mediated by reduced *Ascl1* function. Interestingly, even with this enlarged MGE region, the general patterning of the ventral telencephalon in *Tcf3/12* dcKOs appears to be preserved through normal regional expression of different progenitor markers. However, the generation of appropriate glia and neurons from MGE was severely disrupted in dcKOs with nearly a complete absence of OPCs and an expansion of Nkx2.1 and *Lhx6* expression into the LGE, which is likely the result of the increased number of MGE cells migrating in a tangential manner into the LGE. While the increased MGE phenotype is unique to our *Tcf3/12* dcKOs, other bHLH knockout models have cortical interneuron phenotypes. Ascl1 is required for cortical interneuron production and overexpression promotes cortical interneuron migration [73, 74]. It is possible that increased total Ascl1 + cells in the enlarged MGE of dcKOs produces a gain-of-function effect of Ascl1, leading to increase migration of the expanded MGE cells. Alternatively, *Olig1*^*−/−*^ mice have been shown to overproduce interneurons at the expense of oligodendrocytes from the medial septal progenitor regions and ventral most MGE [62]. The role of *Olig1* in regulating cell fate is consistent with the observations in *Tcf3/12* dcKOs, suggesting that loss of *Tcf3/12* could produce a loss-of-function for Olig1. In fact, Tcf12 was previously identified to be Olig1’s preferred binding partner, while Tcf3 also had some ability to heterodimerize with Olig1 [38]. However, it likely *Olig1* is not alone in mediating all the *Tcf3/12* dcKO phenotypes, since the *Olig1*^*−/−*^ mice do not show the enlarged MGE phenotype or the severe embryonic OPC phenotype in the ventral telencephalon observed in the *Tcf3/12* dcKOs [58, 62]. *Olig2*^*−/−*^ embryos have a very severe OPC generation phenotype in the ventral telencephalon similar to the *Tcf3/12* dcKOs [58, 60, 71]. However, key phenotypic differences remain including that *Olig2*^*−/−*^ embryos do not display the enlarged MGE and show minimal defects in cortical interneuron development from the MGE with only a specific reduction in ChAT + cells in the ventral regions [75–77]. Also, the presence of Olig2 + cells in dcKOs although reduced and the severe reduction of Pdgfrα and Sox10 cells suggests that there may be a defect in OPC expansion or maturation. The similarities between *Olig1* KO, *Olig2* KO, and *Tcf3/12* dcKO defects in oligodendrogenesis, suggest that Tcf3, Tcf12 and Olig1/2 share some common roles during development. Future experiments are required to understand whether there is an in vivo interaction between Tcf3/12 and Olig1/2 or whether these bHLH TFs converge on the same developmental event.

It is not uncommon to observe recovery of OL populations in genetic models that initially perturb these populations [12, 13, 21, 58]. The timing of recovery provides some insight into the *Tcf3/12* dcKO phenotype. OPC production in the telencephalon has distinct spatiotemporal components. OPCs initially are produced by the MGE around E12.5, then are produced by both the MGE and LGE around E15.5, and OPCs generated after E17.5 are produced from the dorsal telencephalon [66, 78]. It is generally accepted that the cortically derived OPCs will replace the majority of MGE derived OPCs to populate the telencephalon [66]. The timing of the OPC recovery observed in this study would be consistent with the production of cortical/dorsal OPCs, indicating *Tcf3* and *Tcf12* may not play an important role in cortical OPC generation unlike ventral (LGE/MGE) OPC generation. However, even after OPC recovery at P7, we observed sustained reductions in the mature OL populations of *Tcf3/12* dcKOs at P7, P21, and P60. Therefore, the recovered OPCs as early as P7 in the dcKOs, appear to be incapable of properly differentiating as late as P60, suggesting that *Tcf3* and *Tcf12* may also be involved in the differentiation of OLs at postnatal stages. In support of this idea, our later recombination studies of *Tcf3/12* using *Olig1*^*Cre/+*^ did not result in an expanded MGE region but still caused a reduction in embryonic OPCs and also mOLs at postnatal stages. This suggests that that *Tcf3* and *Tcf12* also play distinct roles in early embryonic OPC generation and later OL differentiation at postnatal stages.

Our results reveal that *Tcf3* and *Tcf12* play a role in regulating cell fate decisions from the MGE, generating embryonic OPCs, and generating appropriate numbers of OLs at postnatal stages. There remains much that is not understood about class I bHLH TFs and their roles or interactions with class II bHLH TFs. However, this study and the recent work on Tcf4 [38, 72]. have implicated all 3 E-proteins as major regulators during distinct stages of developmental oligodendrogenesis.

## Data Availability

Original data for this study are included in the article. Inquires for data can be directed to the corresponding author.
